# Clinical Prediction in Early Pregnancy of Infants Small for Gestational Age by Customised Birthweight Centiles: Findings from a Healthy Nulliparous Cohort

**DOI:** 10.1371/journal.pone.0070917

**Published:** 2013-08-05

**Authors:** Lesley M. E. McCowan, John M. D. Thompson, Rennae S. Taylor, Robyn A. North, Lucilla Poston, Philip N. Baker, Jenny Myers, Claire T. Roberts, Gustaaf A. Dekker, Nigel A. B. Simpson, James J. Walker, Louise C. Kenny, on behalf of the SCOPE Consortium

**Affiliations:** 1 Department of Obstetrics and Gynaecology, University of Auckland, Auckland, New Zealand; 2 National Women’s, Auckland District Health Board, Auckland, New Zealand; 3 Department of Paediatrics, University of Auckland, Auckland, New Zealand; 4 Division of Women’s Health, Women’s Health Academic Centre, King’s College London and King’s Health Partners, London, United Kingdom; 5 Liggins Institute, University of Auckland, Auckland, New Zealand; 6 Maternal and Fetal Heath Research Centre, University of Manchester, Manchester, United Kingdom; 7 Robinson Institute, University of Adelaide, Adelaide, Australia; 8 Women and Children’s Division, Lyell McEwin Hospital, University of Adelaide, Adelaide, Australia; 9 Section of Obstetrics and Gynaecology, Institute of Biochemical and Clinical Sciences, University of Leeds, Leeds, United Kingdom; 10 INFANT (The Irish Centre for Fetal and Neonatal Translational Research) Department of Obstetrics and Gynaecology, University College, Cork, Ireland; Dartmouth Medical School, United States of America

## Abstract

**Objective:**

Small for gestational age (SGA) infants comprise up to 50% of all stillbirths and a minority are detected before birth. We aimed to develop and validate early pregnancy predictive models for SGA infants.

**Methods:**

5628 participants from SCOPE, a prospective study of nulliparous pregnant women, were interviewed at 15±1 weeks’ gestation. Fetal anthropometry, uterine and umbilical Doppler studies were performed at 20±1 weeks’. The cohort was divided into training (n = 3735) and validation datasets (n = 1871). All-SGA (birthweight <10th customised centile), Normotensive-SGA (SGA with normotensive mother) and Hypertensive-SGA (SGA with mother who developed hypertension) were the primary outcomes. Multivariable analysis was performed using stepwise logistic regression firstly using clinical variables and then with clinical and ultrasound variables. Receiver operator curves were constructed and areas under the curve (AUC) calculated.

**Results:**

633 infants (11.3%) in the whole cohort were SGA; 465 (8.3%) Normotensive-SGA and 165 (3.0%) Hypertensive-SGA. In the training dataset risk factors for All-SGA at 15±1 weeks’ included: family history of coronary heart disease, maternal birthweight <3000 g and 3000 g to 3499 g compared with ≥3500 g, >12 months to conceive, university student, cigarette smoking, proteinuria, daily vigorous exercise and diastolic blood pressure ≥80. Recreational walking ≥4 times weekly, rhesus negative blood group and increasing random glucose were protective. AUC for clinical risk factors was 0.63. Fetal abdominal or head circumference z scores <10^th^ centile and increasing uterine artery Doppler resistance at 20±1 weeks’ were associated with increased risk. Addition of these parameters increased the AUC to 0.69. Clinical predictors of Normotensive and Hypertensive-SGA were sub-groups of All-SGA predictors and were quite different. The combined clinical and ultrasound AUC for Normotensive and Hypertensive-SGA were 0.69 and 0.82 respectively.

**Conclusion:**

Predictors for SGA of relevance to clinical practice were identified. The identity and predictive potential differed in normotensive women and those who developed hypertension.

## Introduction

Approximately 50% of non-anomalous stillborn infants are small for gestational age (SGA) and survivors are at increased risk of neurodevelopmental delay, cerebral palsy [1] and later cardiovascular complications and diabetes [2]. Traditionally, SGA has been defined using population-based birthweight centiles but utilization of customized centiles has enabled identification of additional small babies at higher risk of morbidity and mortality [3], [4].

In routine antenatal care, less than a quarter of all SGA babies are identified before birth [5]. SGA infants who are recognized and well managed before birth have been reported to have a four-fold reduction in perinatal death and severe fetal distress [6]. Utilization of customized antenatal growth charts in routine antenatal care has been associated with improvements in antenatal identification of SGA infants with detection rates of up to 50% [5], [7] but there remains a need for early pregnancy tools which would further increase the level of detection. Development of a reliable early pregnancy risk prediction algorithm enabling identification, monitoring and timely delivery of SGA infants has the potential to reduce both morbidity and mortality in these vulnerable infants. SGA infants can be broadly classified into two main categories with distinct maternal phenotypes: SGA with a normotensive mother (Normotensive-SGA) and SGA where the mother has gestational hypertension, preeclampsia or chronic hypertension (Hypertensive-SGA). We have previously reported that Normotensive- SGA comprises approximately three quarters of all SGA infants born to nulliparous women and that risk factors for Normotensive-SGA and Hypertensive-SGA differ, suggesting that they could be considered as distinct conditions from the perspective of prediction [8].

Our primary aim was to develop clinical risk prediction models for All-SGA, Normotensive-SGA and Hypertensive-SGA infants using clinical data obtained at 15 weeks’ gestation and to determine whether addition of fetal measurements and uterine and umbilical artery Doppler data from the 20 week ultrasound scan improved prediction based on clinical risk alone.

## Materials and Methods

The participants were healthy nulliparous women with singleton pregnancies recruited to the SCOPE (Screening for Pregnancy Endpoints) study between November 2004 and February 2011 in Auckland, New Zealand, Adelaide, Australia, Manchester, Leeds and London, United Kingdom and Cork, Ireland. SCOPE (www.scopestudy.net) was a prospective, multi-centre cohort study with the main aim of developing screening tests to predict preeclampsia, SGA infants and spontaneous preterm birth. Ethical approval was obtained from the relevant institutional ethic committees responsible for human experimentation and all women provided written informed consent. In New Zealand, approval for the SCOPE study was given by Northern Region Ethics Committee on 23/04/2003 reference number: AKX/02/00/364, in Australia by Central Northern Adelaide Health Service Ethics of Human Research Committee on 02/09/2005 reference number: REC 1714/5/Application number 2005082, in London, Leeds and Manchester by the NHS South East Research Ethics Committee, South East Coast Strategic Health Authority, Kent on 19/01/2007 reference number: 06/MRE01/98 and in Ireland by the University College Cork, Teaching Hospital Research Ethics Committee on 06/02/2008 reference number: ECM5(10)05/02/08. Detailed methods have previously been described [9], [10]. The study is registered with Australian New Zealand Clinical Trials Registry ACTRN12607000551493 at https://www.anzctr.org.au.

Women were recruited to the study at 15±1 weeks’ gestation. Those considered at high risk of preeclampsia, SGA or spontaneous preterm birth because of underlying medical conditions (including known pre-existing chronic hypertension on antihypertensive medication or with a blood pressure greater than 160/100 mmHg at 15 weeks’ gestation), three or more previous miscarriages or terminations of pregnancy, or who had received interventions that may modify pregnancy outcome (such as low dose aspirin) were excluded.

Women who agreed to participate were interviewed and examined by a research midwife at 15±1 and 20±1 weeks of gestation and underwent ultrasound examination at 20±1 weeks. Detailed clinical data were collected at each time point, including demographic information, medical history, the woman’s birthweight, previous early pregnancy losses (miscarriage or termination of pregnancy), history of infertility, duration of sexual relationship, gynaecological history and family history of obstetric complications and medical disorders. A family history of metabolic disease was defined as the participant's mother or father having one or more of the following conditions: type 2 diabetes, chronic hypertension, history of cerebrovascular accident or ischaemic heart disease. Current pregnancy data included vaginal bleeding, hyperemesis, dietary information, folic acid and low dose multivitamin supplementation, smoking, alcohol and recreational drug use. A vegetarian diet was defined as no consumption of meat or fish. Binge drinking was defined as drinking 6 or more units of alcohol at one time point. Vigorous exercise was defined as exercise leading to heavy breathing or being out of breath [11] and recreational walking as the number of times per week the woman engaged in walking for recreation or exercise during the previous month. Maternal random glucose (mmol/L) and proteinuria were also measured at 15±1 weeks’ gestation. Proteinuria at 15 weeks’ gestation was defined as a dipstick ≥1+ or spot urine protein: creatinine ratio measurement ≥30 mg/mmol. At the time of interview, data were entered into a secure internet-accessed, auditable database (MedSciNet AB, Sweden).

Ultrasound examination performed at 20±1 weeks’ gestation included fetal anthropometric measurements (biparietal diameter, femur length and head and abdominal circumference) and Doppler waveform analysis of the umbilical and uterine arteries [12]. Mean uterine resistance index (RI) was calculated from the left and right uterine RI. If only a left or right RI was available, this was used as ‘mean RI’ (n = 95). Women who did not have growth measurements or uterine or umbilical artery Doppler assessment performed at 20 weeks were excluded from the analysis of ultrasound factors.

Participants were followed prospectively, with pregnancy outcome data and infant measurements recorded by research midwives, usually within 72 hours of birth.

### Outcome Measures

*All SGA* was defined as birthweight <10^th^ customised centile, where birthweight is adjusted for maternal height, booking weight, ethnicity as well as delivery gestation and infant sex [13]. The primary outcomes were: *Normotensive-SGA* defined as birth of an SGA infant where the mother did not develop hypertension, and *Hypertensive-SGA* defined as birth of an SGA infant where the mother had gestational hypertension, preeclampsia and/or mild chronic hypertension [9].

### Definitions

*Gestational hypertension*: systolic BP≥140 mmHg and/or diastolic BP ≥90 mmHg on at least 2 occasions 4 h apart after 20 weeks’ gestation, but before the onset of labour. *Preeclampsia:* gestational hypertension or postpartum hypertension (as defined above, but developing for the first time after delivery) in association with proteinuria (24 hour urinary protein ≥ 300 mg, or spot urine protein: creatinine ratio ≥ 30 mg/mmol, or urine dipstick protein ≥ 2+) or any multi-system complication of preeclampsia [14], [15], [16]. *Mild chronic hypertension:* a systolic BP of 140–159 mmHg and/or diastolic BP 90–99 mmHg, on more than one reading, first identified at the 15±1 or 20±1 weeks’ gestation SCOPE visit. None of these women had recognised or treated hypertension before the pregnancy. *Non-SGA* referred to all women in the remainder of the cohort who did not have SGA babies. This group included pregnancies with other complications such as spontaneous preterm birth or preeclampsia with a normally grown baby. *Perinatal deaths* included *stillbirths and terminations of pregnancy after 20 weeks’ gestation* [defined as birth of an infant at ≥20 weeks’gestation with no signs of life (n = 33)] and *neonatal deaths* [defined as death of a live born infant in the first 28 days of life (n = 7)]. Antenatal recognition of SGA was defined as SGA being diagnosed on ultrasound before the date of delivery.

### Datasets

**Figure 1:**
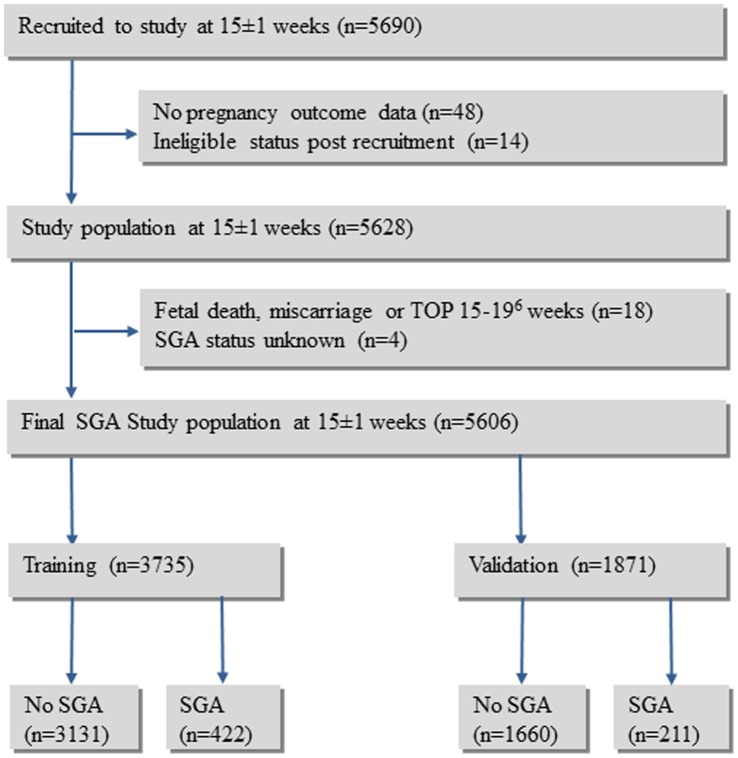
Flow chart of study population.

The final SCOPE cohort for the SGA study population comprised 5606 participants (Figure 1). To allow for model generalization, the data were partitioned into a training set for model fitting and a validation set for empirical validation. Training and validation datasets were generated in a 2∶1 ratio by randomly splitting the total dataset, stratified by the geographical areas of Australasia and Europe. The datasets were checked for major discrepancies in SCOPE centre or rates of SGA between the training and validation sets.

Of the 1055 original and derived variables available to the All-SGA, Normotensive-SGA and Hypertensive-SGA models, 113 were excluded as they were not applicable to prediction. A further 46 variables were excluded as they were not generalizable and 102 variables were excluded as they had >10% missing data due to the variable being added to the database after the initiation of the study, clinical laboratory data or work related variables. Of the remaining 794 variables, univariable analysis for association with All-SGA, Normotensive-SGA and Hypertensive-SGA was performed and 579 All-SGA, 565 Normotensive-SGA and 544 Hypertensive-SGA variables were then excluded as non-significant (P>0.1). Of the remaining available variables [All-SGA (n = 215), Normotensive-SGA (n = 229) and Hypertensive-SGA (n = 250)], the variables in the final models were selected based on known potential risk factors for SGA, ease of collection in the clinical setting and potential applicability to future populations. The deleted variables included those with a low cell count (<5) in the χ^2^ test, lack of *a priori* knowledge regarding SGA prediction or addressed by other variables which were retained in the models. This resulted in 38 All-SGA variables (32 clinical and six 20±1 week ultrasound variables), 26 Normotensive-SGA variables (20 clinical and six 20±1 week ultrasound variables) and 17 Hypertensive-SGA variables (13 clinical and four 20±1 week ultrasound variables) being available to train the three multivariable models (variables listed in Table S1).

The relationship between SGA and the continuous variables available for inclusion in the models was explored using Generalised Additive Models (GAMs) in SAS 9.1 (SAS Institute Inc, Cary, NC) to determine the nature of the relationship and whether variables should be available to the model(s) continuously or categorically. For maternal birthweight, systolic and diastolic blood pressure, and mean uterine artery Doppler resistance index, the relationship with SGA was not linear and these variables were categorised to best reflect the associations in each model.

Among the 15 week clinical variables available to any of the three models, data were complete for 26 and seven variables had data missing for <1.5% of participants. For the remaining variable (maternal birthweight) 5.1% had missing data. Missing continuous data variables were imputed using Expectation Maximization (n = 3) or median (n = 2) and for categorical data variables the mode was used (n = 3).

### Statistical Analysis

Univariable analysis was performed using clinical variables obtained at the 15±1 weeks’ gestation SCOPE interview, and the fetal measurements and Doppler parameters obtained at the 20±1 weeks’ gestation ultrasound scan using the unimputed database. The comparison group for All-SGA was Not-SGA, for ‘Normotensive-SGA’ was ‘Not Normotensive-SGA’ (which comprised all women in the remainder of the cohort who did not have ‘Normotensive-SGA’ including those with ‘Hypertensive-SGA’), and for ‘Hypertensive-SGA’ the comparison group was ‘Not Hypertensive-SGA’ (which comprised all women in the remainder of the cohort who did not have ‘Hypertensive –SGA’ including those with ‘Normotensive-SGA’). Other pregnancy pathologies were therefore included in the comparison groups.

Student’s t-test or Wilcoxon rank sum tests were used for comparison of continuous variables. Categorical variables were compared using chi-square or Fisher’s exact test. Multivariable analysis was performed using both forward and backward stepwise logistic regression firstly using clinical variables only, and then with clinical and ultrasound variables, to compare All-SGA, Normotensive-SGA and Hypertensive-SGA with their respective comparison groups. All final models were the same when the forward and backward methods were used. For the multivariable analyses using the 15 week clinical variables, imputed databases were used but for the ultrasound analyses women with missing data were not included in the analysis. A significance level of less than or equal to 0.05 was used in multivariable analyses. Receiver operator curves were produced for all multivariable models along with the associated area under the curve (AUC) and screening test characteristics reported at a 25%, 10% and 5% false positive rate [17]. SAS 9.1(SAS Institute Inc, Cary, NC) was used for all statistical analyses.

## Results

Five thousand six hundred and ninety healthy nulliparous women with singleton pregnancies were recruited to the SCOPE (Screening for Pregnancy Endpoints) study between November 2004 and February 2011 in Auckland, New Zealand, Adelaide, Australia, London, Leeds and Manchester, United Kingdom and Cork, Ireland and follow up was complete in 98.9% of participants (Figure 1) (STROBE Statement: Table S2).

To allow for model generalization, the data were divided into a training set for model fitting (n = 3735) and a validation set (n = 1871) (Table 1). Of the 422 (11%) infants in the training database who were SGA by customized centiles, 313 (8%) were born to normotensive mothers (Normotensive-SGA), 109 (3%) to hypertensive mothers (Hypertensive-SGA) (Table 2). In the validation database, 211 (11%) infants were SGA of whom 152 (8%) were Normotensive-SGA and 59 (3%) Hypertensive-SGA. Comparing training and validation sets, amongst the Hypertensive-SGA pregnancies, 44 (40%) and 26 (44%) women respectively had preeclampsia; 63 (58%), and 33 (56%) had gestational hypertension; 2 (2%) and none had mild chronic hypertension alone. Antenatal recognition of SGA was not significantly different between normotensive mothers with SGA infants (109, 23%) and hypertensive mothers with SGA infants (46, 28%), p = 0.35.

**Table 1:** Study population characteristics at 15±1 weeks’ and pregnancy outcome.

	Training Cohort			Validation Cohort		
	SGA	Not-SGA	P value	SGA	Not-SGA	P value
	N = 422	N = 3313		N = 211	N = 1660	
**Maternal Characteristics**						
Maternal age (y)	28.6 (5.8)	28.6 (5.4)	0.94	28.5 (5.7)	28.9 (5.5)	0.40
Ethnicity			0.30			0.83
White	373 (88%)	2994 (91%)		191 (91%)	1487 (90%)	
Maori or Pacific Islander*	10 (2%)	61 (2%)		3 (1%)	39 (2%)	
Asian or Indian	23 (6%)	179 (5%)		12 (6%)	88 (5%)	
Other†	16 (4%)	79 (2%)		5 (2%)	46 (3%)	
Primigravid	312 (74%)	2568 (78%)	0.09	154 (73%)	1285 (77%)	0.15
Single	48 (11%)	304 (9%)	0.15	31 (15%)	154 (9%)	0.009
No paid employment	75 (18%)	465 (14%)	0.04	43 (20%)	243 (15%)	0.03
<12 years education	168 (40%)	1223 (37%)	0.25	90 (43%)	635 (38%)	0.22
Smoking status			<0.0001			<0.0001
Non smoker	286 (68%)	2538 (77%)		142 (67%)	1283 (77%)	
Ceased smoking before15 wks	55 (13%)	444 (13%)		29 (14%)	225 (14%)	
Current smoker	81 (19%)	331 (10%)		40 (19%)	152 (9%)	
BMI category			0.02			0.06
<20.0	36 (8%)	221 (7%)		14 (7%)	131 (8%)	
20.0–24.9	189 (45%)	1702 (51%)		87 (41%)	816 (49%)	
25.0–29.9	117 (28%)	908 (27%)		69 (33%)	478 (29%)	
≥30	80 (19%)	482 (15%)		41 (19%)	235 (14%)	
Maternal birthweight (g)	3163 (540)	3329 (559)	0.0001	3184(499)	3319 (523)	<0.001
	N = 395	N = 3144		N = 197	N = 1583	
Systolic BP (mmHg)	108 (11)	107 (10)	0.004	109 (11)	107 (11)	0.01
Diastolic BP (mmHg)	66 (9)	65 (8)	0.01	67 (8)	65 (8)	0.002
**Pregnancy Outcome**						
Hypertensive Pregnancy‡	109 (26%)	409 (12%)	<0.0001	59 (28%)	183 (11%)	<0.0001
Birthweight (g)	2641 (557)	3500 (516)	<0.0001	2627 (614)	3512 (483)	<0.0001
Gestational age at delivery (wks)	39.0 (3.4)	39.7 (2.0)	<0.0001	38.5 (3.6)	39.8 (1.8)	<0.0001
Customised birthweight centile	4.7 (3)	53.0 (26)	<0.0001	4.7 (3)	53.3 (26)	<0.0001
Total preterm births (<37 wks)	54 (13%)	177 (5%)	<0.0001	40 (19%)	80 (5%)	<0.0001
Admitted to neonatal unit*	82 (19%)	340 (10%)	<0.0001	61 (29%)	164 (10%)	<0.0001
Perinatal deaths	7 (1.7%)	15 (0.5%)	<0.0001	5 (2%)	1 (0.1%)	<0.0001

Results expressed as N (%) or mean (SD) as appropriate;

* includes 44 Maori and 27 Pacific Islanders in the training and 27 Maori and 15 Pacific Islanders in the validation set;

† includes 15 Australian Aborigines in the training and 8 in the validation set.

‡ Hypertensive pregnancy defined as preeclampsia, gestational hypertension or mild chronic hypertension.

**Table 2:** Maternal characteristics and pregnancy outcome amongst Normotensive and Hypertensive-SGA pregnancies.

	Normotensive-SGA	Hypertensive-SGA	P value
	N = 313 (11.4%)	N = 109 (2.9%)	P value
**Maternal Characteristics**			
Maternal age (years)	28.4 (5.8)	29.3 (5.8)	0.18
Ethnicity			0.50
White	273 (87%)	100 (92%)	
Maori or Pacific Islander*	8 (2%)	2 (2%)	
Indian	9 (3%)	4 (3%)	
Asian	9 (3%)	1 (1%)	
Other†	14 (5%)	2 (2%)	
Primigravid	231 (74%)	81 (74%)	1.0
Socioeconomic index	41 (17)	38 (16)	0.19
Smoker at 15±1 weeks	65 (21%)	16 (15%)	0.20
BMI category (kg/m^2^)			<0.0001
<20.0	6 (2%)	0 (0%)	
20.0–24.9	179 (57%)	40 (37%)	
25.0–29.9	81 (26%)	36 (33%)	
>30	47 (15%)	33 (30%)	
Participant’s birthweight (grams)	3166 (551)	3178 (463)	0.79
	N = 292	N = 101	
Systolic BP 15±1 weeks	107 (11)	113 (12)	<0.0001
Diastolic BP 15±1 weeks	64 (8)	70 (9)	<0.0001
**Pregnancy Outcome**			
Birthweight (grams)	2686 (531)	2506 (611)	0.004
Gestational age at delivery (weeks)	39.3 (3.5)	38.3 (3.3)	0.01
Customised birthweight centile	5.0 (2.9)	3.8 (2.8)	<0.0001
Total preterm births (<37 weeks)	27 (9%)	27 (25%)	<0.0001
Admitted to neonatal unit	44 (14%)	38 (35%)	<0.0001
Perinatal deaths	5 (2%)	2 (2%)	

Results expressed as N (%), mean (SD);

* includes 4 Maori and 6 Pacific Islanders;

† includes 5 Australian Aborigines.

**Figure 2:**
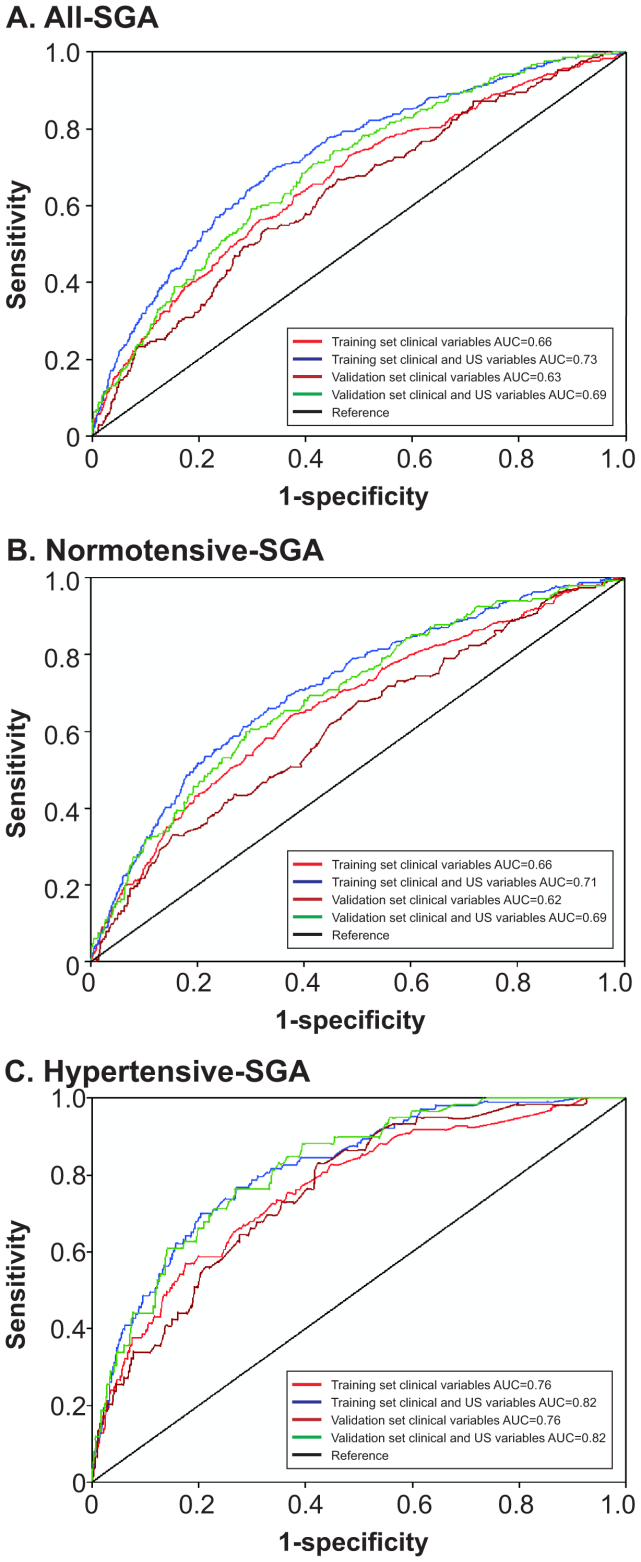
Receiver operating characteristics curves based on training and validation models of clinical risk factors at 15 weeks’ and ultrasound factors at 20 weeks’ gestation. A. All SGA B. Normotensive SGA C. Hypertensive SGA.

Clinical variables associated with prediction of All-SGA in the training dataset model (Table 3) included: family history of coronary heart disease, maternal birthweight <3000 g, and between 3000 g and 3499 g compared with ≥3500 g, taking >12 months to conceive, attending university, cigarette smoking, the presence of proteinuria, daily vigorous exercise and diastolic BP≥80. Recreational walking ≥4 times weekly, rhesus negative blood group, and increasing random glucose were protective. The AUC for clinical risk factors in the training dataset was 0.66. Fetal abdominal or head circumference Z score <10^th^ centile and increasing mean uterine artery Doppler resistance indices at 20 weeks were also associated with increased risk, and addition of these ultrasound parameters to clinical risk factors resulted in an AUC of 0.73. In the validation dataset, the AUC for clinical risk factors alone was 0.63 and after addition of the ultrasound parameters was 0.69 (Figure 2A).

**Table 3:** Univariable and multivariable comparisons of clinical and ultrasound risk factors for All SGA by customised birthweight centiles.

			Training Cohort		
			N = 3735		N = 3541
Risk Factors	SGA	Non SGA	Univariable Odds Ratio (95% CI)†	Multivariable Odds Ratio (95% CI)	Multivariable Odds Ratio (95% CI)††
	N = 422	N = 3313			
**15 week data**				**AUC = 0.66**	**AUC = 0.73**
Family history of coronary heart disease (father)	72 (17.1)	402 (12.1)	1.49 (1.13 to 1.96)	1.46 (1.10 to 1.93)	1.63 (1.22, 2.19)
Maternal birthweight (grams)*	3163 (539)	3329 (559)			
<3000	138 (35%)	749 (24%)	2.18 (1.66 to 2.86)	2.09 (1.60 to 2.74)	1.98 (1.49, 2.63)
3000–3499	157 (40%)	1212 (38%)	1.53 (1.17 to 1.99)	1.56 (1.21 to 2.02)	1.56 (1.19, 2.05)
≥3500	100 (25%)	1183 (38%)	1.00	1.00	1.00
>12 months to conceive	89 (21%)	526 (16%)	1.42 (1.10 to 1.82)	1.48 (1.14 to 1.91)	1.36 (1.03, 1.78)
Currently attending university	20 (5%)	88 (3%)	1.82 (1.12 to 3.00)	1.91 (1.14 to 3.19)	1.87 (1.08, 3.24)
Currently smoking	81 (19%)	331 (10%)	2.14 (1.64 to 2.80)	2.15 (1.63 to 2.83)	2.13 (1.59, 2.84)
Proteinuria	28 (7%)	126(4%)	1.80 (1.18 to 2.74)	1.61 (1.04 to 2.50)	1.57 (0.99, 2/49)
Vigorous exercise ≥1 x/day	12 (3%)	29 (0.9%)	3.33 (1.69 to 6.57)	3.78 (1.87 to 7.66)	3.22 (1.43, 7.29)
Diastolic BP≥80 mmHg	39 (9%)	176 (5%)	1.82 (1.26 to 2.61)	1.84 (1.27 to 2.68)	1.82 (1.21, 2.73)
*Recreational walking ≥4 x/week*	*69 (17*%*)*	*701 (21*%*)*	*0.73 (0561 to 0.96)*	*0.72 (0.55 to 0.96)*	*0.73 (0.55, 0.98)*
*Rhesus negative blood group*	*41(10*%*)*	*512 (15*%*)*	*0.59 (0.42 to 0.82)*	*0.58 (0.41 to 0.81)*	*0.60 (0.42, 0.85)*
*Random glucose per unit increase*	*5.2 (0.9)*	*5.3 (1.0)*	*0.87 (0.78 to 0.98)*	*0.85 (0.76 to 0.95)*	*0.84 (0.75, 0.95)*
**20 week scan data** ††	**N = 401**	**N = 3174**			
Head circumference z score <10^th^ centile**	66 (17%)	267 (8%)	2.14 (1.60 to 2.87)		1.79 (1.30 to 2.46)
Abdominal circumference z score <10^th^ centile***	64 (16%)	278 (9%)	1.98 (1.47 to 2.65)		1.73 (1.25 to 2.39)
Average uterine resistance index	0.61 (0.10)	0.56 (0.10)			
<0.5	57 (14%)	844 (26%)	1.0		1.0
0.5 to 0.59	111 (28%)	1259 (40%)	1.31 (0.94 to 1.82)		1.31 (0.93 to 1.84)
0.6 to 0.69	151 (38%)	752 (24%)	2.97 (2.16 to 4.10 )		2.80 (2.01 to 3.90)
0.7 to 0.79	65 (16%)	259 (8%)	3.72 (2.54 to 5.44)		3.63 (2.44 to 5.39)
0.8 to 1.0	17 (4%)	60 (2%)	4.20 (2.30 to 7.66)		4.56 (2.45 to 8.48)

Data are mean (SD) number (%) as appropriate, italics indicate variable is protective.

* missing data numbers for woman’s birthweight (SGA n = 27, no SGA 169 missing.

** missing data numbers for head circumference z score <10^th^ centile (SGA n = 2, no SGA n = 23 missing).

*** missing data numbers for abdominal circumference z score <10^th^ centile (SGA n = 1, no SGA n = 12 missing).

† Unimputed data was used for univariable analyses.

†† Unimputed ultrasound data was added to the multivariable models derived from 15 week imputed data.

Clinical variables associated with prediction of Normotensive-SGA in the training dataset model (Table 4) were a sub-group of those included in the All-SGA model. The AUC for clinical risk factors in the training dataset was 0.66. Fetal abdominal or head circumference Z score <10^th^ centile and increasing mean uterine artery Doppler resistance indices at 20±1 weeks’ were again associated with increased risk, and addition of these ultrasound parameters to clinical risk factors resulted in an AUC of 0.71. In the validation dataset, the AUC for clinical risk factors alone was 0.62 and with the addition of the ultrasound parameters was 0.69 (Figure 2B).

**Table 4:** Univariable and multivariable comparisons of clinical and ultrasound risk factors for Normotensive-SGA by customised birthweight centiles.

			Training Cohort		
			N = 3735		N = 3541
Risk Factors	NT SGA	Non NT SGA	Univariable Odds Ratio (95% CI) †	Multivariable Odds Ratio (95% CI)	Multivariable Odds Ratio (95% CI) ††
	N = 313	N = 3422			
**15 week data**				**AUC = 0.66**	**AUC = 0.71**
Maternal birthweight (grams)*	3166 (551)	3323 (558)			
<3000	105 (36%)	782 (24%)	2.05 (1.51 to 2.78)	2.01 (1.49 to 2.72)	1.88 (1.37 to 2.58)
3000–3499	110 (37%)	1259 (39%)	1.33 (0.99 to 1.80)	1.38 (1.03 to 1.85)	1.37 (1.01 to 1.85)
≥3500	79 (27%)	1204 (37%)	1.0	1.00	1.00
Currently attending university	19 (6%)	89 (3%)	2.42 (1.46 to 4.03)	2.52 (1.49 to 4.25)	2.50 (1.43 to 4.37)
Currently smoking	65 (21%)	347 (10%)	2.32 (1.73 to 3.12)	2.29 (1.69 to 3.09)	2.22 (1.62 to 3.04)
Vigorous exercise ≥1 x/day	10 (3%)	31 (0.9%)	3.63 (1.76 to 7.48)	3.97 (1.87 to 8.41)	3.91 (1.6 to 9.07)
*Recreational walking ≥4 x/week*	*45 (15*%*)*	*725 (21*%*)*	*0.63 (0.45 to 0.87)*	*0.61 (0.44 to 0.85)*	*0.58 (0.41 to 0.83)*
*Rhesus negative blood group*	*25 (8*%*)*	*528 (15*%*)*	*0.48 (0.31 to 0.72)*	*0.48 (0.31 to 0.73)*	*0.51 (0.33 to 0.78)*
*Random glucose per unit increase*	*5.2 (0.9)*	*5.3 (1.0)*	*0.86 (0.76 to 0.97)*	*0.85 (0.75 to 0.96)*	*0.84 (0.73 to 0.96)*
**20 week scan data** ††	**N = 298**	**N = 3277**			
Head circumference z score <10^th^ centile**	50 (17%)	283 (9%)	2.13 (1.53 to 2.95)		1.70 (1.19 to 2.43)
Abdominal circumference z score <10^th^ centile***	50 (17%)	292 (9%)	2.06 (1.49 to 2.86)		1.78 (1.25 to 2.54)
Average uterine resistance index	0.61 (0.10)	0.57 (0.10)			
<0.5	40 (13%)	861 (26%)	1.0		1.0
0.5 to 0.59	94 (32%)	1276 (39%)	1.59 (1.08 to 2.32)		1.56 (1.06 to 2.30)
0.6 to 0.69	110 (37%)	793 (24%)	2.99 (2.05 to 4.34 )		2.73 (1.86 to 4.00)
0.7 to 1.0	54 (18%)	347 (11%)	3.35 (2.19 to 5.14)		3.21 (2.07 to 4.97)

Data are mean (SD) number (%) as appropriate, italics indicate variable is protective.

* missing data numbers for woman’s birthweight (SGA n = 19, no SGA 178 missing.

** missing data numbers for head circumference z score <10^th^ centile (SGA n = 2, no SGA n = 23 missing).

*** missing data numbers for abdominal circumference z score <10^th^ centile (SGA n = 1, no SGA n = 12 missing).

† Unimputed data was used for univariate analyses.

†† Unimputed ultrasound data was added to the multivariable models derived from 15 week imputed data.

NT SGA = birth of an SGA infant where the mother did not develop hypertension.

Non NT SGA = all women in the remainder of the cohort who did not have Normotensive-SGA pregnancies.

Variables identified as predictors of Hypertensive-SGA in the training database were predominantly a sub-group of All-SGA predictors: taking greater than 12 months to conceive, a family history of metabolic syndrome or coronary heart disease, low fruit consumption, binge drinking, overweight and obesity, systolic BP≥120 mm Hg and diastolic BP≥80 mm Hg (Table 5). As with Normotensive-SGA, maternal birthweight <3000 g and between 3000 g and 3499 g was a significant predictor. The AUC for the clinical variables identified at 15±1 weeks’ for prediction of Hypertensive-SGA was 0.76. Addition of mean uterine artery Doppler resistance indices resulted in an improvement of the model (AUC = 0.82). There was no change in the AUC in the validation cohort (0.76 for clinical alone and 0.82 for clinical plus ultrasound) (Figure 2C).

**Table 5:** Univariable and multivariable comparisons of clinical and ultrasound risk factors for Hypertensive-SGA by customised birthweight centiles.

			Training Cohort		
			N = 3735		N = 3541
Risk Factors	HTSGA	Non HTSGA	Univariable Odds Ratio (95% CI) †	Multivariable Odds Ratio (95% CI)	Multivariable Odds Ratio (95% CI) ††
	N = 109	N = 3626			
**At 15 weeks**				**AUC = 0.76**	**AUC = 0.82**
>12 months to conceive	28 (26%)	587 (16%)	1.79 (1.15 to 2.78)	1.70 (1.08 to 2.67)	1.64 (1.02 to 2.64)
Family history of metabolic syndrome (mother)	44 (40%)	936 (26%)	1.95 (1.32 to 2.87)	1.67 (1.11 to 2.51)	1.59 (1.04 to 2.44)
Family history of coronary heart disease (father)	25 (23%)	449 (12%)	2.11 (1.33 to 3.33)	1.77 (1.09 to 2.85)	1.85 (1.12 to 3.05)
Low fruit consumption in the past month‡	58 (53%)	1413 (39%)	1.78 (1.22 to 2.61)	1.94 (1.30 to 2.89)	1.98 (1.30 to 3.00)
Any binge drinking during pregnancy	40 (37%)	841 (23%)	1.92 (1.29 to 2.86)	2.14 (1.42 to 3.23)	1.97 (1.28 to 3.04)
Maternal birthweight (grams) *	3179 (463)	3314 (561)			
<3000	33 (33%)	854 (25%)	2.32 (1.33 to 4.04)	2.10 (1.22 to 3.62)	2.20 (1.24 to 3.90)
3000 to 3499	47 (47%)	1322 (39%)	2.14 (1.27 to 3.59)	2.09 (1.26 to 3.44)	2.22 (1.31 to 3.77)
≥3500	21 (21%)	1262 (36%)	1.0	1.0	1.0
Body Mass Index (kg/m^2^)					
<25	40 (37%)	2108 (58%)	1.0	1.0	1.00
25 to 29.9	36 (33%)	989 (27%)	1.92 (1.22 to 3.03)	1.47 (0.92 to 2.36)	1.46 (0.89 to 2.39)
≥30.0	33 (30%)	529 (15%)	3.29 (2.05 to 5.26)	1.96 (1.18 to 3.27)	2.00 (1.18 to 3.38)
Systolic blood pressure ≥120 mmHg	38 (35%)	478 (13%)	3.53 (2.35 to 5.29)	1.97 (1.21 to 3.20)	2.03 (1.21 to 3.40)
Diastolic blood pressure ≥80 mmHg	25 (23%)	190 (5%)	5.38 (3.37 to 8.61)	2.87 (1.65 to 5.02)	2.66 (1.45 to 4.86)
**20 week ultrasound scan data** ††	**N = 103**	**N = 3472**			
Average uterine Doppler resistance index	0.64 (0.12)	0.57 (0.10)			
<0.5	17 (16%)	884 (25%)	1.00		1.00
0.5 to 0.59	17 (16%)	1353 (39%)	0.65 (0.33, 1.29)		0.63 (0.31, 1.25)
0.6 to 0.69	41 (40%)	862 (25%)	2.47 (1.39, 4.39)		2.29 (1.27, 4.13)
0.7 to 0.79	18 (18%)	306 (9%)	3.06 (1.56, 6.01)		2.72 (1.35, 5.47)
0.8 to 1.0	10 (10%)	67 (2%)	7.76 (3.42, 17.62)		7.83 (3.32, 18.45)

Data are mean (SD) number (%) as appropriate,

* missing data numbers for woman’s birthweight (SGA n = 8, no SGA n = 188 missing).

† Unimputed data was used for univariate analyses.

†† Unimputed ultrasound data were added to the multivariable models derived from imputed 15 week data.

‡ <1serving of fruit/day.

HT SGA = birth of an SGA infant where the mother had gestational hypertension, preeclampsia and/or mild chronic hypertension.

Non HT SGA = all women in the remainder of the cohort who did not have Hypertensive-SGA pregnancies.

The test characteristics for prediction of All-SGA, Normotensive-SGA and Hypertensive-SGA at 5%, 10% and 25% false positive rates in the validation dataset are shown in table 6.

**Table 6:** Prediction of All-SGA, Normotensive-SGA and Hypertensive-SGA based on clinical risk factors at 15±1 weeks’ combined with ultrasound factors at 20±1 weeks’ gestation in a nulliparous cohort using cut-offs at 5%, 10%, and 25% false positive rates.

No (%) with abnormal test result	Sensitivity	Specificity	PPV	NPV	Positive Likelihood ratio	Negative Likelihood ratio
**All-SGA Validation cohort**						
114 (6.2%)	15 (11 to 20)	95 (94 to 96)	27 (20 to 36)	90 (89 to 91)	3.0 (2.0 to 4.4)	0.90 (0.84 to 0.94)
218 (11.8%)	26 (21 to 32)	90 (89 to 91)	24 (19 to 30)	91 (90 to 91)	2.6 (1.9 to 3.4)	0.83 (0.76 to 0.89)
517 (27.9%)	52 (45 to 58)	75 (74 to 76)	21 (18 to 23)	93 (92 to 94)	2.1 (1.7 to 2.4)	0.65 (0.56 to 0.74)
**Normotensive-SGA Validation cohort**						
109 (5.9%)	16 (10 to 23)	95 (94 to 96)	21 (14 to 30)	93 (92 to 94)	3.1 (2.0 to 4.8)	0.89 (0.83 to 0.95)
217 (11.7%)	31 (23 to 39)	90 (88 to 91)	21 (16 to 27)	94 (92 to 95)	3.1 (2.3 to 4.1)	0.77 (0.69 to 0.86)
505 (27.3%)	53 (45 to 61)	75 (73 to 77)	15 (12 to 19)	95 (94 to 96)	2.1 1.8 to 2.5)	0.63 (0.53 to 0.74)
**Hypertensive SGA Validation cohort**						
111 (6.0%)	34 (22 to 47)	95 (94 to 96)	18 (12 to 27)	98 (97 to 98)	6.7 (4.4 to 10.0)	0.70 (0.58 to 0.84)
206 (11.1%)	44 (31 to 58)	90 (88 to 91)	13 (9 to 18)	98 (97 to 99)	4.4 (3.2 to 6.0)	0.62 (0.50 to 0.78)
492 (26.6%)	71 (58 to 82)	75 (73 to 77)	9 (6 to 11)	99 (98 to 99)	2.8 (2.4 to 3.4)	0.38 (0.26 to 0.57)

## Discussion

In the large international prospective SCOPE study we have identified a number of factors present in the first half of pregnancy which are associated with later development of SGA. The models detailed above may be considered as the first step towards the development of a personalised algorithm for prediction of SGA in healthy nulliparous women to which biomarkers will be added as the next step. Although All-SGA was undertaken initially, the rationale for considering the prediction of Normotensive and Hypertensive-SGA separately include the largely different phenotypes and the more severe perinatal outcomes in Hypertensive-SGA. Metabolic syndrome features such as obesity, higher blood pressure and family history of metabolic disease are predominant in the Hypertensive-SGA model whereas in Normotensive-SGA factors such as smoking and vigorous exercise are incorporated.

Predictors for All-SGA reflect a combination of the Normotensive and Hypertensive-SGA sub-groups. The area under the ROC curve for prediction of All-SGA infants in the validation database using clinical risk factors recorded at the 15±1 weeks’ gestation was 0.63 which increased to 0.69 with addition of fetal growth and mean uterine artery Doppler parameters from the 20±1 weeks’ anatomy scan. At a false positive rate of 10% and 25%, 26% and 52% of All-SGA pregnancies respectively were identified in the validation database. This is comparable to detection rates reported in routine clinical practice and with the use of customised antenatal growth charts respectively [7]. We defined SGA as birthweight less than the tenth customised centile, which is better associated with pathological fetal growth than SGA defined as birthweight less than the tenth population centile [3], [18], [19]. However a proportion of these small babies will inevitably still be constitutionally small and healthy.

Not surprisingly the predictors for All-SGA we identified in this study are very similar to the risk factors that we reported after the first 3500 women had been recruited to the SCOPE study [9]. Lower maternal birthweight and cigarette smoking are well described risk factors for SGA infants [9]. As has previously been reported women who undertook daily vigorous exercise had a greater than three-fold increase in risk of SGA [9], perhaps mediated by reduced uterine blood flow [9], [20]. Interestingly women who undertook moderate exercise (defined as recreational walking ≥four times weekly) had a 27% reduction in risk. This latter novel finding with regard to protection from SGA, supports the American College of Obstetrics and Gynaecology Guidelines recommendation that low to moderate intensity exercise should be encouraged in pregnancy [21]. The biological mechanism is unclear but may be related to improved cardiovascular function and insulin sensitivity in pregnant women who exercise [22], [23]. We also re-confirmed that Rhesus negative maternal blood group is a non-modifiable protective factor associated with an approximate 40% reduction in risk of SGA. The gene encoding the Rhesus factor lies within a group of genes on chromosome 1 which are associated with transportation mechanisms into cells for several molecules including zinc, glucose and ammonia [24] which may explain the protective effect on fetal growth found in this and our previous study [9]. Additionally we found that increasing maternal random glucose at 15±1 weeks’ was protective for SGA which is consistent with findings from the HAPO study which demonstrated a continuous relationship between increasing maternal glucose and birthweight [25].

Consistent with data from our previous publication [8] the large majority of SGA infants in this study (73.8%) were born to mothers who remained normotensive in pregnancy and hence there is substantial overlap between All-SGA and Normotensive-SGA predictors and similar performance of the two models with area under the ROC curve for prediction of Normotensive-SGA infants in the validation database using clinical risk factors of 0.62 increasing to 0.69 with addition of ultrasound parameters from the 20±1 weeks’ anatomy scan. At a false positive rate of 10% and 25%, 31% and 53% of Normotensive-SGA pregnancies respectively were identified in the validation database. As for All-SGA prediction models, these rates are comparable to detection rates in current clinical practice [7].

The performance of our model for Normotensive-SGA is comparable to a previous report for prediction of SGA pregnancies in mothers without preeclampsia which combined uterine artery Doppler, blood pressure and biomarkers at 11–13 weeks’ gestation and identified 50% of later SGA pregnancies [26]. We have recently reported an AUC of 0.9 for prediction of SGA at 15 weeks’in a matched case (n = 40) control (n = 40) sub-group of SCOPE participants without preeclampsia (and therefore predominantly Normotensive-SGA) using a combination of 19 metabolites including sphingolipids, phospholipids, carnitines, and fatty acids [27]. We are therefore optimistic that improved prediction of Normotensive-SGA will be achievable in the next phase of SCOPE research program, in which we plan to combine clinical risk factors with measurement in all participants of multiple placental, endothelial and cardiovascular biomarkers, representing different potential pathways in the pathogenesis of SGA [28]. In line with this approach, a recent systematic review of biomarkers for predicting fetal growth restriction has demonstrated poor predictive ability of single biomarkers and also recommended combinations of clinical factors with biophysical parameters and multiple biomarkers in future studies [29].

With the exception of dietary factors, the predictors for Normotensive-SGA we identified in this study are very similar to the risk factors that we reported after the first 3500 women had been recruited to the SCOPE study [9]. SCOPE participants in the training and validation data-sets in this study were stratified by the geographical areas of Australasia and Europe whereas in the earlier study the large majority of participants were from Auckland, New Zealand (58%) and Adelaide, Australia (32%). The balanced geographic distribution in the current study may explain why the previously identified risk factors of pre-pregnancy fruit and vegetable intake, which were very low in the Adelaide participants, were no longer associated with Normotensive-SGA in this study [30]. Currently attending University was associated with All-SGA and Normotensive-SGA.We are unable to explain this relationship which we believe is unlikely to be causal and more likely to be related to residual confounding. It is unlikely to be a surrogate for higher socioeconomic status which should reduce the risk of SGA [28].

Prediction of Hypertensive-SGA infants by our algorithm using clinical characteristics, achieved an AUC of 0.76 which was increased to 0.82 by the addition of ultrasound variables from the 20 week scan in the validation model. At a false positive rate of 10% and 25%, 44% and 71% of Hypertensive-SGA pregnancies were identified in the validation database. A detection rate of 71% using this model is considerably better than routine practice [10] and is two and a half times greater than the 28% detection rate of hypertensive SGA in the current study. This is an important sub-group of SGA pregnancies to predict antenatally given the increased rates of preterm delivery and more severe fetal growth restriction we have demonstrated, both of which are associated with increased rates of long term adverse neurological outcomes [31].

Other factors, including smoking [32], [33], alcohol intake [34], maternal birthweight [35], increasing body mass index [33], increasing blood pressure [36] and increasing uterine artery Doppler indices [37] previously identified as being associated with SGA were also included in our model of Hypertensive-SGA. Furthermore, the variables included in our Hypertensive-SGA prediction model are very similar to those which we recently reported as predictors of preeclampsia [10]. This is interesting as less than half of Hypertensive-SGA pregnancies in our cohort had preeclampsia (42%) and the majority group had gestational hypertension (57%) with only 1% with chronic hypertension. This finding suggests that predictors for gestational hypertension associated with SGA pregnancies and preeclampsia may be very similar [38].

Several previous studies which have aimed to predict late pregnancy complications such as preeclampsia and SGA have used heterogeneous populations of mixed parity which included women with well-established risk factors [26], [36] limiting generalizability of findings. In the SCOPE study our goal is to predict late pregnancy complications in healthy nulliparous women. The reasons for this are several-fold: nulliparous women comprise >40% of births: in the United Kingdom [39], Ireland [40], Australia [41] and New Zealand [42], they have significantly higher rates of preeclampsia, SGA and spontaneous preterm birth than multiparous women, they do not have a past obstetric history to guide risk selection, and screening results are more likely to be generalizable to other groups of healthy nulliparous women. We also elected to develop the SCOPE screening tests based primarily on data collected at 15±1 weeks’ of gestation rather than in the first trimester. The reasons for this approach are that preventative therapy with low dose aspirin is effective for reduction of preeclampsia and SGA when initiated at 16 and up to 20 weeks’ gestation [43], [44], more women have booked for antenatal care and biomarker discrimination in particular with PlGF has been shown to improve between samples obtained in the first trimester and 15–16 weeks’ [45].

For each SGA predictive model there was a 5–6% increase in performance with addition of fetal size and/or Doppler indices from the 20±1 weeks’ anatomy scan. We are not advocating incorporating uterine artery Doppler as a routine component of the 20 week anatomy scan as the performance of our algorithms is currently not good enough for application in clinical practice. We will be able to comment further on the utility of 20 week Doppler studies after biomarkers are added to the clinical algorithms in the next phase of our SCOPE research.

The urgent need for improved prediction of SGA pregnancies is highlighted by the fact that only one quarter (24.5%) of all SGA infants were identified before birth in our cohort. Improved detection accompanied by good management has been associated with reduced mortality and severe morbidity in SGA infants [6] and is a high priority in antenatal care. It was disappointing that antenatal identification was not significantly improved in Hypertensive-SGA pregnancies (27.9% in Hypertensive-SGA vs 23.4% in Normotensive-SGA p = 0.35). It is well described that preeclampsia is associated with a two to three fold increase in risk whereas gestational hypertension is associated with an approximately 50% increase in risk of SGA [8], [46]. Women with hypertensive complications in late pregnancy should therefore have a growth scan unless delivery is to be undertaken forthwith, in which case the fetus should be monitored carefully during the delivery process.

The strengths of our study included its prospective design, excellent follow up of a large cohort (98.7%), and real-time data monitoring procedures that helped ensure the quality of the data. The comparison group for our predictive models was all other women. For example for Normotensive-SGA the comparison group was all women without Normotensive-SGA (which included Hypertensive-SGA and other pregnancy complications) and for Hypertensive-SGA it was women without Hypertensive-SGA, which included those with Normotensive-SGA. Some previous studies aiming to predict preeclampsia or SGA have used women with uncomplicated pregnancies as the referent group [47], [48]. This artificially enhances test performance and also does not reflect the real clinical situation where at the beginning of pregnancy late pregnancy outcome is unknown.

Our rich database contains variables covering all known and hypothesised risk factors based on biological plausibility for SGA infants. A limitation imposed by this study design and our wealth of data is that we could not pre-select a limited number of variables based on prior knowledge and biological plausibility for our logistic regression models.

### Conclusions

In a large cohort of healthy nulliparous women we report combinations of clinical and ultrasound variables obtained in the first half of pregnancy associated with later development of SGA. Future addition of multiple biomarkers to our clinical and ultrasound algorithms may enable reliable risk prediction, especially of Hypertensive-SGA pregnancies. If successful this will enable monitoring and timely delivery of these SGA infants with the potential to reduce both morbidity and mortality.

## Supporting Information

Table S1 **Variables available to SGA, Normotensive SGA or Hypertensive SGA models.**
(DOCX)

Table S2 **STROBE Statement - Checklist of items that should be included in reports of cohort studies.**
(DOCX)
