# Supplementary material for: Clinical Prediction in Early Pregnancy of Infants Small for Gestational Age by Customised Birthweight Centiles: Findings from a Healthy Nulliparous Cohort
Source: PLoS One. 2013 Aug 5;8(8):e70917. doi: 10.1371/journal.pone.0070917 (PMC3733741; doi:10.1371/journal.pone.0070917)
Supplement: Table S1 — Variables available to SGA, Normotensive SGA or Hypertensive SGA models. (DOCX) [file pone.0070917.s001.docx]

**Table S1: Variables available to SGA, Normotensive SGA or Hypertensive SGA models**

| **Variable Name** | **Variable categories** | **Comments** | **Missing** | **Imputation type** | **All-SGA**  **N=38** | **Norm-SGA**  **N=26** | **Hyper-SGA**  **N=17** |
| --- | --- | --- | --- | --- | --- | --- | --- |
| Maternal age (years) | 1: <25y  2: 25-34y  3: ≥ 35y |  | Nil |  | ✓ | ✓ |  |
| Currently attending university | No Yes |  | Nil |  | ✓ | ✓ |  |
| Paid employment | No Yes | Included full and part-time paid employment at 15 weeks. | Nil |  | ✓ | ✓ |  |
| Participant’s birthweight | 1: <3000  2: 3000 to 3499  3: >3500 | Data was self-reported, but women were asked to bring their ‘birth record’ if kept at home. Birthweight was confirmed from this birth record in 77%. Imputed with expectation maximum likelihood estimation (EM). | 5.1% | EM | ✓ | ✓ | ✓ |
| Primigravida | No Yes |  | Nil |  | ✓ |  |  |
| Months of sexual relationship with partner ≤3 months duration. | No Yes |  | 0.2% | Median | ✓ | ✓ |  |
| Any pregnancy loss <10weeks with same partner | No Yes | Any previous termination or miscarriage <=10wks gestation or an ectopic pregnancy with same man who has fathered the current pregnancy. | Nil |  | ✓ |  |  |
| History of infertility | No Yes | History of infertility defined as >=12 months of regular intercourse without contraception and conception has not occurred or if partner is known to be sterile. 0.2% were unknown and were included in the ‘No’ category. | Nil |  | ✓ |  | ✓ |
| Hypertension on combined oral contraception (OC) | No Yes | Participant had been told on more than one occasion that her blood pressure was elevated by her health care worker when on combined OC or had ceased the OC because of raised blood pressure. | Nil |  | ✓ |  | ✓ |
| Participant’s mother had a history of gestational hypertension | No Yes | Mother of woman developed gestational hypertension defined as new onset hypertension in second half of pregnancy and known to not have proteinuria. | Nil |  | ✓ |  |  |
| Participant’s mother had a history of preterm birth | No Yes | Participant's mother had a history of recurrent preterm births (included both spontaneous and iatrogenic preterm births) | Nil |  | ✓ |  |  |
| Participant’s mother had a history of low birth weight baby | No  Yes | Mother of woman had one or more low birth weight babies defined as <2500g. | Nil |  | ✓ | ✓ |  |
| Participant’s mother had a history of metabolic disease | No  Yes | Mother of woman had one or more of type 2 diabetes, chronic hypertension, CVA and IHD. | Nil |  | ✓ |  | ✓ |
| Participant’s father has coronary heart disease | No  Yes | Father of woman has had a heart attack, coronary heart disease, coronary bypass, angioplasty or angina. If father had coronary heart disease=Yes; no paternal history of coronary heart disease or no paternal history available=No. | Nil |  | ✓ |  | ✓ |
| Vegetarian | No  Yes | Woman’s diet did not include meat or fish. | Nil |  | ✓ |  | ✓ |
| Non-oily fish intake pre-pregnancy | No  Yes | History of eating any non-oily fish including shellfish in the month prior to pregnancy | Nil |  | ✓ |  |  |
| Fruit intake pre-pregnancy | 1: <1/day  2: >1/day | The average number of servings of fruit in the month prior to conception. | Nil |  | ✓ | ✓ | ✓ |
| Fruit intake at 15 weeks | 1: <1/wk  2: 1-6x/wk  3: >=1/day | The average number of servings of fruit in the month prior to the 15 week visit. | Nil |  | ✓ |  | ✓ |
| Green leafy vegetable intake pre-pregnancy | 1: <1/wk 2: 1-6x/wk  3: >=1/day | The average number of servings of green leafy vegetables in the month prior to conception. | Nil |  | ✓ | ✓ |  |
| High oily fish at 15 weeks | No  Yes | History of eating high amounts of oily fish (defined as three or more servings per week) in the month prior to the 15 week visit. Included salmon, tuna, trout, sardines, herrings, mackerel. | Nil |  |  | ✓ |  |
| Multivitamin intake at 15±1 weeks | No  Yes | Any multivitamin intake at the 15±1 week visit. Women were asked to bring these to the 15 week visit. | 0.3% | Mode | ✓ | ✓ |  |
| Smoking at 15±1 weeks | No  Yes | Women who continued to smoke at the time of the15 week visit. | Nil |  | ✓ | ✓ |  |
| Alcohol intake at 15±1 weeks | 1: No alcohol in pregnancy  2: Quit alcohol prior to 15 weeks  3: Continuing to drink alcohol at 15 weeks | Alcohol consumption at 15 weeks included any consumption of alcohol in the week prior to the 15 week visit. | Nil |  |  | ✓ |  |
| Binge Alcohol in pregnancy | No Yes | Any binge alcohol consumed in pregnancy up to the time of the 15±1 weeks. Binge defined as the consumption of > 6 units of alcohol at one time point. | Nil |  | ✓ |  | ✓ |
| Other drug use at 15±1 weeks | No Yes | Consumed/inhaled/injected recreational drugs other than cigarettes and alcohol. Included binge drinking. | Nil |  | ✓ | ✓ |  |
| Systolic blood pressure at 15±1 weeks | 1: < 120  2: >120 | Second measurement of systolic blood pressure. GAMs in SAS was used to determine the shape of the relationship with SGA as opposed to assuming a linear relationship and 2 categories were then created. | Nil |  | ✓ |  | ✓ |
| Diastolic blood pressure at 15±1 weeks | 1: < 80  2: >80 | Second measurement of diastolic blood pressure. GAMs in SAS was used to determine the shape of the relationship with SGA as opposed to assuming a linear relationship and 2 categories were then created. | Nil |  | ✓ |  | ✓ |
| Body mass index (BMI) at 15±1 weeks | 1: <20  2: 20-24.9  3: 25-29.9  4: >30 | weight (kg) / height^2^ (m). GAMs in SAS was used to determine the shape of the relationship with SGA as opposed to assuming a linear relationship and 4 categories were then created. | Nil |  | ✓ | ✓ | ✓ |
| Head circumference at 15±1 weeks | 1: < 80  2: >80 | Maternal head circumference measured, in centimetres, GAMs in SAS was used to determine the shape of the relationship with SGA as opposed to assuming a linear relationship and 2 categories were then created. Imputed with expectation maximum likelihood estimation (EM). | 0.2% | EM | ✓ | ✓ |  |
| Random glucose at 15±1 weeks | Continuous | mmol/L; imputed with expectation maximum likelihood estimation (EM).. | 1.3% | EM | ✓ | ✓ |  |
| Proteinuria at 15±1 weeks | No Yes | Defined as 1+ on dipstick or spot urine protein creatinine ratio (PCR) measurement ≥30 mg/mmol. | 0.6% | Mode dipstick  Median PCR | ✓ | ✓ | ✓ |
| Recreational walking at 15±1 weeks | <4 x /wk  >4 x /wk | Engaged in any walking for recreation or exercise in the last month. | 0.4% | Mode | ✓ | ✓ |  |
| Vigorous exercise at 15±1 weeks | No Yes | Engaged in any daily exercise leading to heavy breathing or being puffed. | 0.4% | Mode | ✓ | ✓ |  |
| Rhesus factor negative | No Yes |  | Nil |  | ✓ | ✓ |  |
| Head circumference z score <10^th^ centile at 20 week scan | No Yes | Fetal head circumference (adjusted for gestational age using multiples of the median) <10^th^centile |  |  | ✓ | ✓ | ✓ |
| Abdominal circumference z score <10^th^ centile at 20 week scan | No Yes | Fetal abdominal circumference (adjusted for gestational age using multiples of the median) <10^th^centile |  |  | ✓ | ✓ |  |
| Femur length z score <10^th^ centile at 20 week scan | No Yes | Fetal femur length (adjusted for gestational age using multiples of the median) <10^th^centile |  |  | ✓ | ✓ |  |
| Umbilical artery resistance index (RI) at 20 week scan | 1: <0.7  2: 0.7-0.79  3: 0.8-1.0 | Umbilical artery Resistance Index (RI) measured using Doppler ultrasound at 19-21w 3 categories created. | Nil |  | ✓ | ✓ | ✓ |
| Mean uterine artery RI at 20 week scan | 1: <0.5  2: 0.5 to 0.59  3: 0.6 to 0.69  4: 0.7 to 0.79  5: 0.8 to 1.0 | Mean uterine RI was calculated from the right and left uterine artery RI. Five categories were created for All-SGA and Hypertensive-SGA and four categories (<0.5/0.5 to 0.59/0.6 to 0.69/0.7 to 1.0) for Normotensive-SGA. | Nil |  | ✓ | ✓ | ✓ |
| Bilateral notches at 20 week scan | No Yes | Diastolic notches in the Doppler waveforms of both the right and left uterine arteries. Reference notch photos provided to ultrasonographers. | Nil |  | ✓ | ✓ | ✓ |
